# Molecular Insight Into the Therapeutic Potential of Long Non-coding RNA-Associated Competing Endogenous RNA Axes in Alzheimer’s Disease: A Systematic Scoping Review

**DOI:** 10.3389/fnagi.2021.742242

**Published:** 2021-11-25

**Authors:** Hani Sabaie, Nazanin Amirinejad, Mohammad Reza Asadi, Abbas Jalaiei, Yousef Daneshmandpour, Omidvar Rezaei, Mohammad Taheri, Maryam Rezazadeh

**Affiliations:** ^1^Molecular Medicine Research Center, Tabriz University of Medical Sciences, Tabriz, Iran; ^2^Department of Medical Genetics, Faculty of Medicine, Tabriz University of Medical Sciences, Tabriz, Iran; ^3^Department of Biology, Faculty of Sciences, Shahid Bahonar University of Kerman, Kerman, Iran; ^4^Skull Base Research Center, Loghman Hakim Hospital, Shahid Beheshti University of Medical Sciences, Tehran, Iran; ^5^Institute of Human Genetics, Jena University Hospital, Jena, Germany

**Keywords:** Alzheimer’s disease, antisense oligonucleotides, competing endogenous RNA, long non-coding RNA, miRNA sponge

## Abstract

Alzheimer’s disease (AD) is a heterogeneous degenerative brain disorder with a rising prevalence worldwide. The two hallmarks that characterize the AD pathophysiology are amyloid plaques, generated via aggregated amyloid β, and neurofibrillary tangle, generated via accumulated phosphorylated tau. At the post-transcriptional and transcriptional levels, the regulatory functions of non-coding RNAs, in particular long non-coding RNAs (lncRNAs), have been ascertained in gene expressions. It is noteworthy that a number of lncRNAs feature a prevalent role in their potential of regulating gene expression through modulation of microRNAs via a process called the mechanism of competing endogenous RNA (ceRNA). Given the multifactorial nature of ceRNA interaction networks, they might be advantageous in complex disorders (e.g., AD) investigations at the therapeutic targets level. We carried out scoping review in this research to analyze validated loops of ceRNA in AD and focus on ceRNA axes associated with lncRNA. This scoping review was performed according to a six-stage methodology structure and PRISMA guideline. A systematic search of seven databases was conducted to find eligible articles prior to July 2021. Two reviewers independently performed publications screening and data extraction, and quantitative and qualitative analyses were conducted. Fourteen articles were identified that fulfill the inclusion criteria. Studies with different designs reported nine lncRNAs that were experimentally validated to act as ceRNA in AD in human-related studies, including *BACE1-AS*, *SNHG1*, *RPPH1*, *NEAT1*, *LINC00094*, *SOX21-AS1*, *LINC00507*, *MAGI2-AS3*, and *LINC01311*. The *BACE1-AS*/*BACE1* was the most frequent ceRNA pair. Among miRNAs, *miR-107* played a key role by regulating three different loops. Understanding the various aspects of this regulatory mechanism can help elucidate the unknown etiology of AD and provide new molecular targets for use in therapeutic and clinical applications.

## Introduction

Alzheimer’s disease (AD) is a progressive neurodegenerative disorder (NDD) and is a form of dementia that triggers difficulties with memory, thinking, and behavior (Kang et al., 2020). Based on the Alzheimer’s Association, AD makes up about approximately 60–80% of dementia cases. Currently, worldwide, 50 million people are living with AD and other dementias. AD incidence doubles every 5 years after age 65 (Fan et al., 2020). Symptoms generally develop slowly and aggravate with time. From a hereditary point of view, AD is a heterogeneous polygenic condition. The condition has been categorized into two groups according to age-onset: early-onset AD (EOAD) and late-onset AD (LOAD). LOAD also called sporadic AD (SAD), is the most common type of dementia. AD is a multifactorial condition as a result of interactions between the susceptible genes and environmental factors (Rezazadeh et al., 2019). Genes play an important role in AD. The heritability of LOAD is 58–79%, while it is more than 90% in EOAD. The genetic association has helped us to understand the etiology of AD. Over 50 loci are currently associated with AD. These findings highly suggest that AD is a complex disease (Sims et al., 2020).

### Alzheimer’s Disease Pathogenesis

The pathophysiology of AD is defined by the accumulation of β-amyloid peptide (Aβ) in the brain, as well as hyperphosphorylated and cleaved structures of the microtubule-associated protein tau. It is known that metabolic dysfunction of Aβ precursor protein (APP) and abnormal tau protein phosphorylation (Kang et al., 2020) or maybe their interaction with each other (Busche and Hyman, 2020) results in senile plaques and neurofibrillary tangles (NFTs) formation. According to biochemical, behavioral, and genetic research, the pathologic development of the neurotoxic Aβ peptide resulting from serial APP proteolysis is a critical step in AD development. Moreover, APP is metabolized rapidly and in a highly complicated manner by groups of sequential secretases, including β-site APP-cleaving enzyme 1 (BACE1), γ-secretase, and the ADAM family as α-secretases. Regarding tau proteolysis, this process is crucial in neurodegeneration and the tau aggregation process. Tau is a microtubule-associated protein that is predominantly produced in neurons and is encoded by the Microtubule-Associated Protein Tau (*MAPT*) gene. Intracellular tau is sometimes hyperphosphorylated, resulting in hazardous oligomers and aggregates visible as NFTs (Kang et al., 2020). Aside from the tau and amyloid theory about AD, several additional ideas have been proposed, namely inflammatory reactions, oxidative stress, mitochondrial failure, and cholinergic hypothesis (Li et al., 2021). Although there are numerous hypotheses about AD pathogenesis, the actual triggers and ideal therapy strategies are still elusive.

### Antisense Therapeutics

For therapeutic development, RNA is a novel target with numerous advantages, including: (Kang et al., 2020) it is applicable to a majority of RNAs in the cells, such as non-coding RNAs (ncRNAs), (Fan et al., 2020) genetic discoveries can directly be translated to drug discovery, and drug discovery would be more rapid and efficient. Whereas other promising progress has been made in the discovery of small-molecule medicines that affect RNA activity, antisense oligonucleotides (ASOs) provide a far straightforward technique (Bennett et al., 2019). ASOs are synthetic oligonucleotides or oligonucleotide analogs with lengths ranging from 12 to 30 nucleotides (nt) and are engineered for binding to RNA through Watson-Crick base pairing. ASOs can be constructed to bind to both protein-coding RNAs (mRNAs) and ncRNAs, including microRNAs (miRNAs) or long non-coding RNAs (lncRNAs). After ASOs bind with the RNAs of interest, antisense medicine can alter the activity of the RNAs in different ways (Bennett and Swayze, 2010; Crooke et al., 2018).

### Long Non-coding RNAs

NcRNAs are classified into two types based on their lengths: short ncRNAs (less than 200 nt) and long ncRNAs (more than 200 nt). lncRNAs range in size from 200 nt to more than 100 kb and often lack a clear open reading frame. LncRNAs, resembling protein-coding genes, are widely transcribed by RNA polymerase II and are frequently post-transcriptionally changed by 5′ capping, 3′ polyadenylation, and RNA splicing processes. However, lncRNAs differ from protein-coding genes in that they possess shorter lengths and exhibit poorer sequence conservation among species (Quinn and Chang, 2016). Many biological functions rely on lncRNAs, including RNA transcription, translation, chromatin and DNA modifications, mRNA stability, and pre-RNA splicing (Li et al., 2021).

### Long Non-coding RNAs and mRNA Stability

The rate of mRNA synthesis and degradation determines its steady-state level; hence, the degradation of mRNA is an important factor in controlling gene expression. Previous research has shown that instability of mRNAs that encode synaptic transmission proteins leads to synaptic function loss in AD pathogenesis (Alkallas et al., 2017). Furthermore, in AD patients, the mRNA degradation rate of AD risk genes is abnormal (Beyer et al., 2009). It has been shown that lncRNAs have a role in AD pathogenesis via regulating mRNA stability (Li et al., 2021).

### Competing Endogenous RNA Hypothesis

**FIGURE 1:**
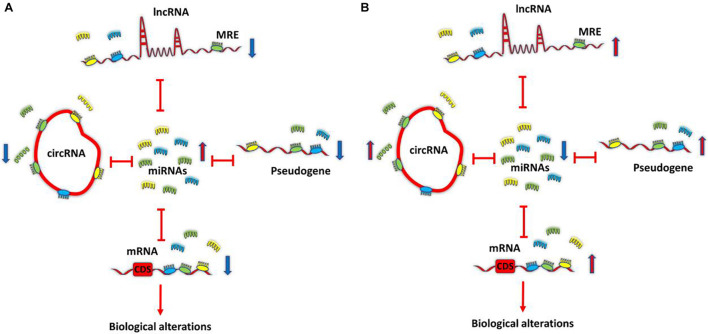
Competing endogenous RNA (ceRNA) model. All transcriptome components, such as long non-coding RNAs (lncRNAs), circRNAs, pseudogenes, and mRNAs that share common MRE, can function as ceRNAs and can co-regulate each other by sponging shared miRNAs. Differentially expressed transcripts can lead to ceRNA dysregulation and biological alterations. ceRNA. **(A)** Down-regulation of ceRNAs increases the amounts of free miRNAs, thereby repressing target expression. **(B)** Conversely, up-regulation of ceRNAs reduces free miRNAs abundance, thereby derepressing target expression. Competing endogenous RNA; circRNA, circular RNA; lncRNA, long non-coding RNA; miRNA, microRNA; MRE, miRNA response element.

A new mechanism of interaction between RNAs, called ceRNA, is proposed by Pier Paolo Pandolfi’s group in 2011. This hypothesis suggests that cross-talk between RNAs, both coding RNAs and ncRNAs (such as lncRNAs, circRNAs: circular RNAs, and pseudogenes) through miRNA complementary sequences called miRNA response elements (MREs) builds a large-scale regulatory network throughout the transcriptome. Based on the ceRNA hypothesis, if two RNA transcripts regulate each other via a ceRNA mediated mechanism, the expression levels of these two RNA transcripts would be negatively correlated with the levels of target miRNAs, and the expression levels of these two RNA transcripts would be positively associated with each other (Salmena et al., 2011). Figure 1 demonstrates the most simplified ceRNA model. Studies of ceRNA interactions are according to the prediction of target RNA transcripts using multiple software programs (Shuwen et al., 2018) such as StarBase (Li et al., 2014), TargetScan (Agarwal et al., 2015), PicTar (Krek et al., 2005), and StarScan (Liu et al., 2015). These predictions are based on identifying the same MRE within multiple RNA sequences. As the precision of prediction programs is unclear (Amirkhah et al., 2015) due to insufficient raw data for algorithms to reference, the prediction results must still be validated (Shuwen et al., 2018).

Over the last few years, several studies have verified the ceRNA theory. It is well known that the disruption of the equilibrium of ceRNA cross-talk can play a role in various diseases (Sen et al., 2014). So far, the ceRNA mechanisms have been further studied in the field of cancer (Yang et al., 2016). During the last 4 years, the study in the field of NDDs has begun systematically, and significant improvements have been produced. Among NDDs, the ceRNA interactions in AD have been studied to a greater extent (Cai and Wan, 2018). Since 2017, there have been increasing studies to identify the genome-wide ceRNA networks in AD using bioinformatics prediction (Wang et al., 2017; Zhang et al., 2017; Sekar et al., 2018; Wang Z. et al., 2018; Yu et al., 2018; Ma N. et al., 2019; Zhang Y. et al., 2019; Zhou et al., 2019; Ma et al., 2020; Xu and Jia, 2020). Since interaction networks of ceRNAs are multifactorial, they might be advantageous in investigations of complicated diseases, like AD, in terms of therapeutic targets only by targeting one of them, the levels of several disease-related RNAs change at once (Moreno-García et al., 2020).

### Aim of Study

In this study, we performed a scoping review to analyze validated ceRNA loops in AD. Our focus was on lncRNA-associated ceRNA axes that underlie AD pathophysiology and could potentially be therapeutic targets.

## Methods

The strategy for this review was according to the scoping review structure recommended by Arksey and O’Malley (2005) and later improved by Levac et al. (2010). This consists of five distinct steps: (1) identifying the research question, (2) identifying relevant studies, (3) study selection, (4) charting the data, and (5) collating, summarizing, and reporting results. An optional sixth step in the scoping review, consultation, was not used in our study. This review was also well guided by the Preferred Reporting Items for Systematic Reviews and Meta-Analyses Extension for Scoping Reviews (PRISMA-ScR) Checklist (Tricco et al., 2018).

### Identifying the Research Question

This study focused on mapping the current literature on lncRNA-associated ceRNA loops in AD. To address this aim, we sought to answer the following question: Precisely what is known from existing literature about lncRNA-associated ceRNA regulatory axes in AD?

### Identifying Relevant Studies

An initial limited search of PubMed and Embase was performed, and then the keywords in the title and abstract were assessed, as well as the index terms that were utilized in the articles. A second search was performed across PubMed, Embase, Scopus, Web of Science, and Cochrane databases according to specific search tips of each database without any restriction, using keywords, MeSH, or Emtree terms recognized from the primary search. The search strategies for PubMed and Embase are shown in Appendix I. Additionally, searches were also carried out in two gray (i.e., difficult to locate or unpublished) literature databases: Google Scholar and ProQuest. The last search was performed on July 10, 2021. We also examined the reference lists of the relevant literature and review articles for additional sources.

### Selecting Studies

The included studies fulfilled the following criteria: (1) explicitly discussing the lncRNA-associated ceRNA axes in AD, (2) written in English, and (3) be original research. The exclusion criteria were (1) studies of non-AD or unspecified dementia, (2) studies that did not use human specimens or cell lines, and (3) studies that did not use molecular techniques to validate the components of the ceRNA loop. The title and abstract of articles were first independently screened by three reviewers (HS, NA) for eligibility according to the above criteria. The full texts of the remaining articles were evaluated, and articles going to fulfill the eligibility criteria were included in the final data analysis. Any disagreements were solved through discussion or with a third reviewer (MR) if required.

### Charting the Data

Three reviewers (HS and NA) independently extracted data into a predesigned charting form in Microsoft Excel. It provided details about the first author, year of publication, origin, type of study, cell line(s), human samples, methods, ceRNAs, shared miRNA(s), and key findings.

### Collating, Summarizing, and Reporting the Results

We performed quantitative and qualitative analyses. For the quantitative part, we provided a descriptive numerical summary of the characteristics of the included articles. For the qualitative analysis, we provided a narrative review of the existing information addressing our earlier mentioned research question, focusing on the importance of results in the broader framework as suggested by Levac et al. (2010).

## Results

### Search Results

**FIGURE 2:**
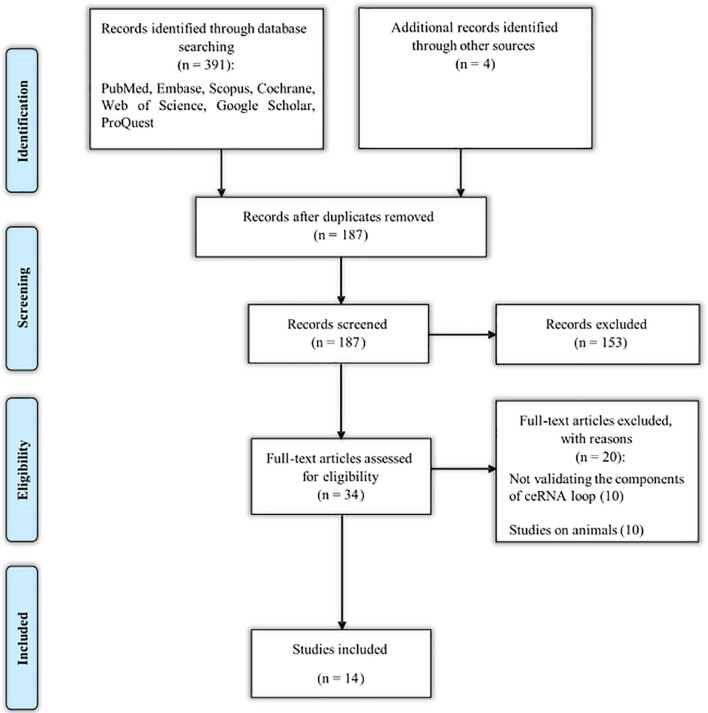
Search strategyflow chart based on the PRISMA flow diagram.

The different steps of finding eligible studies are shown in the flow chart in Figure 2. A total of 395 articles were identified from different sources, of which 208 were duplicates. One hundred and fifty-three articles were excluded for irrelevance. The full texts of the remaining 34 articles were evaluated, and 20 more articles were also excluded because they did not validate the components of the ceRNA loop using molecular techniques (Wang et al., 2017; Yu et al., 2018; Zhou et al., 2019; Ma et al., 2020; Xu and Jia, 2020; Huaying et al., 2021; Lu et al., 2021; Ou et al., 2021; Tang et al., 2021; Zhang et al., 2021) and did not use human specimens or cell lines (Jiang et al., 2016; Cai et al., 2017; Wang J. et al., 2018; Wang X. et al., 2018; Ma P. et al., 2019; Zhao et al., 2019; Li L. et al., 2020; Li X. et al., 2020; Yue et al., 2020; Zhou et al., 2020). Lastly, a total of 14 eligible articles remained (Faghihi et al., 2010; Ke et al., 2019; Wang et al., 2019; Zeng et al., 2019; Zhu et al., 2019; Gao et al., 2020; Ge et al., 2020; Gu et al., 2020, 2021; He W. et al., 2020; Yan et al., 2020; Yue et al., 2020; Fan et al., 2021; Zhang and Wang, 2021).

### Study Characteristics

The characteristics of the included studies are summarized in Table 1. Almost all studies have been published since 2019. All but one study (Faghihi et al., 2010) was conducted in China. Eleven studies examined the ceRNA regulatory loop on human cell lines (Ke et al., 2019; Wang et al., 2019; Zeng et al., 2019; Zhu et al., 2019; Gao et al., 2020; Ge et al., 2020; Gu et al., 2020, 2021; Yan et al., 2020; Yue et al., 2020; Fan et al., 2021) and three on both human specimens and cell lines (Faghihi et al., 2010; He W. et al., 2020; Zhang and Wang, 2021). All studies with human specimens had a case-control design. One study used brain samples (Faghihi et al., 2010), one used plasma samples (He W. et al., 2020), and one used serum samples (Zhang and Wang, 2021).

**TABLE 1:** Characteristics of studies included in the scoping review.

First author	Year of publication	Origin	Type of study	Cell line(s)	Human samples	Methods	ceRNAs	Shared miRNA(s)	Key findings	References
Faghihi et al.	2010	United States	Case-control, cell culture	HEK293T	Brain	Bioinformatics analysis, RT-PCR, high-throughput sequencing, enzyme complementation assay, luciferase activity assay	*BACE1-AS* and *BACE1*	*miR-485-5p*	*BACE1-AS* and to a lower level *BACE1* were up-regulated, and the expression of *miR-485-5p* was decreased in AD patients	Faghihi et al., 2010
Ke et al.	2019	China	Cell culture	SH-SY5Y, SK-N-SH, HEK293T	–	Bioinformatics analysis, qRT-PCR, cell viability assay, immunocytochemistry, cell apoptosis assay, western blot, luciferase activity assay, RIP assay	*NEAT1* and targets of *miR-107*	*miR-107*	*NEAT1* had been up-regulated in Aβ-treated cell lines. It was pointed out as a sponge of *miR-107*. Knockdown of *NEAT1* attenuated Aβ induced inhibition of viability and promotion of apoptosis and p-tau levels. *miR-107* was down-regulated, and it reversed Aβ-induced injury when overexpressed in Aβ-treated cells.	Ke et al., 2019
Wang et al.	2019	China	Cell culture	SH-SY5Y, HEK293T	–	Bioinformatics analysis, RNA interference, qRT-PCR, western blot, MTT assay, flow cytometry, MMP assay, caspase-3 activity assay, luciferase reporter assay	*SNHG1* and *KREMEN1*	*miR-137*	Neuronal cell damage caused by Aβ increased the expression of *SNHG1*. *SNHG1* acted as a sponge for *miR-137*, and the knockdown of *SNHG1* applied its neuronal protective effects through inhibiting *KREMEN1*.	Wang et al., 2019
Zeng et al.	2019	China	Cell culture	HEK293 T, SH-SY5Y, U251	–	Bioinformatics analysis, RIP assay, western blot, real-time PCR, RNA interference, dual-luciferase assay	*BACE1-AS* and *BACE1*	*miR-29b-3p*/*miR-107*/*miR-124-3p*/*miR-485-5p*/*miR-761*	The overexpression of *BACE1-AS* repressed the miRNAs that target *BACE1* and increased Aβ levels. Knockdown of *BACE1-AS* increased the expressions of these miRNAs and reduced the expression of *BACE1.*	Zeng et al., 2019
Zhu et al.	2019	China	Cell culture	hCMEC/D3, HEK293T	–	Bioinformatics analysis, real-time PCR, microarrays, TEER assays, western blot, immunofluorescence assays, luciferase reporter assay, RIP assay	*LINC00094* and *SH3GL2*	*miR-224-5p*/*miR-497-5p*	*LINC00094* was dramatically increased in the Aβ-incubated BBB model. *LINC00094* reduction inhibited the expression of *SH3GL2* through up-regulation of *miR-224-5p*/*miR-497-5p* and finally resulted in alleviated permeability of BBB in the AD microenvironment.	Zhu et al., 2019
Gao et al.	2020	China	Cell culture	SK-N-SH, CHP 212	–	Bioinformatics analysis, CCK8 assay, qRT-PCR, flow cytometry, western blot, ELISA, RNA interference, dual-luciferase reporter assay, RIP assay	*SNHG1* and *ZNF217*	*miR-361-3p*	*SNHG1* expression was positively regulated by Aβ and negatively regulated by resveratrol. It sponged *miR-361-3p* and promoted cell injury via *SNHG1*/*miR-361-3p*/*ZNF217* axis. *SNHG1* knockdown could reverse the promotion effect of Aβ on cell injury.	Gao et al., 2020
He et al.	2020	China	Case-control, cell culture	SK-N-SH, SK-N-AS	Plasma	Bioinformatics analysis, RNA interference, qRT-PCR, cell proliferation assay, flow cytometry, western blot, dual-luciferase reporter assay	*BACE1-AS* and targets of *miR-214-3p*	*miR-214-3p*	Up-regulation of *BACE1-AS* and down-regulation of *miR-214-3p* was found in the cell models treated with Aβ and isoflurane, as well as plasma samples of AD patients. *BACE1-AS* sponged *miR-214-3p* and exacerbated isoflurane-induced neurotoxicity. Both BACE1-AS reduction and miR-214-3p up-regulation reversed the suppression of proliferation and the facilitation of apoptosis and autophagy of Aβ-treated induced by isoflurane.	He W. et al., 2020
Xu et al.	2020	China	Cell culture	SH-SY5Y, SK-N-SH	–	Bioinformatics analysis, RNA interference, qRT-PCR, cell viability assay, flow cytometry, western blot, dual-luciferase reporter assay, RIP assay	*SOX21-AS1* and targets of *miR-107*	*miR-107*	Up-regulation of *SOX21-AS1* and down-regulation of *miR-107* were seen in Aβ-treated cell models. *SOX21-AS1* sponged *miR-107*, and its silencing reduced Aβ-induced neuronal damage.	Yue et al., 2020
Yan et al.	2020	China	Cell culture	SH-SY5Y	–	Bioinformatics analysis, RNA interference, qRT-PCR, western blot, FISH, luciferase reporter assay	*LINC00507* and *MAPT*/ *TTBK1*	*miR-181c-5p*	The Up-regulation of *LINC00507* was seen in the AD model. *LINC00507* sponged *miR-330-5p* and caused the up-regulation of *MAPT*/*TTBK1*. This axis regulates tau hyperphosphorylation via P25/P35/GSK3β signaling pathway.	Yan et al., 2020
Gu et al.	2020	China	Cell culture	SK-N-SH	–	MTT assay, flow cytometry, caspase-3 activity, qRT-PCR, western blot, dual-luciferase reporter assay	*RPPH1* and *WNT1*	*miR-122*	Over-expressed *RPPH1* activated Wnt/β-catenin signaling to ameliorate amyloid-β induced neuronal apoptosis through direct miR-122.	Gu et al., 2020
Ge et al.	2020	China	Cell culture	HPN, SK-N-SH, HEK297T	–	Bioinformatics analysis, MTT assay, cytotoxicity assay, apoptosis assay, western blot, qRT-PCR, RNA interference, dual-luciferase reporter assay	*BACE1-AS* and targets of *miR-132-3p*	*miR-132-3p*	The combined therapy of Berberine treatment with *BACE1-AS* depletion protected neuronal cells against Aβ25–35 through the *BACE1-AS*/*miR-132-3p* axis.	Ge et al., 2020
Gu et al.	2021	China	Cell culture	SH-SY5Y	–	MTT assay, qRT-PCR, fluo-4 NW calcium assay, apoptosis assay, western blot, dual-luciferase reporter assay	*RPPH1* and *PKM2*	*miR-326*	*RPPH1* directly targeted *miR-326*. Thereby its inhibitory impact on the expression of *PKM2* was counteracted, helping to attenuate endoplasmic reticulum stress and apoptosis caused by Ab25-35.	Gu et al., 2021
Zhang and Wang	2021	China	Case-control, cell culture	SH-SY5Y, BV2, HEK293	Serum	RNA interference, dual-luciferase reporter assay, qRT-PCR, MTT assay, ELISA	*MAGI2-AS3* and *BACE1*	*miR-374b-5p*	The *MAGI2-AS3* expression was increased, and *miR-374b-5p* expression was declined in cell models exposed to Aβ25–35. The *miR-374b-5p* and *MAGI2-AS3* serum levels in patients with AD showed a negative correlation; however, they were correlated with the severity of the disorder. *MAGI2-AS3* reduction enhanced neuronal viability and attenuated neuroinflammation in AD. The neurotoxicity induced by Aβ is regulated by *MAGI2-AS3*/*miR-374b-5p* axis.	Zhang and Wang, 2021
Fan et al.	2021	China	Cell culture	SH-SY5Y	–	Bioinformatics analysis, qRT-PCR, RNA interference, apoptosis assay, proliferation assay, autophagy assay, endogenous APP assay, dual-luciferase reporter assay	*LINC01311* and targets of *miR-146a-5p*	*miR-146a-5p*	*LINC01311* was downregulated, whereas miR-146a-5p was up-regulated in the AD cell model. *LINC01311* up-regulation and *hsa-miR-146a-5p* downregulation led to the protection of the apoptosis induced by AB1-42, autophagy, decelerated proliferation, and accumulation of APP in cell models of AD.	Fan et al., 2021

*Aβ, amyloid-beta; BBB, blood–brain barrier; AD, Alzheimer’s disease; CCK8, cell counting kit 8; ceRNA, competing endogenous RNA; circRNA, circular RNA; ELISA, enzyme-linked immunosorbent assay; FISH, fluorescence in situ hybridization; lncRNA, long non-coding RNA; miRNA, microRNA; MMP, mitochondrial membrane potential; qPCR, quantitative polymerase chain reaction; qRT-PCR, quantitative reverse transcription-polymerase chain reaction; RIP, RNA immunoprecipitation; RT-PCR, reverse transcription-polymerase chain reaction.*

**FIGURE 3:**
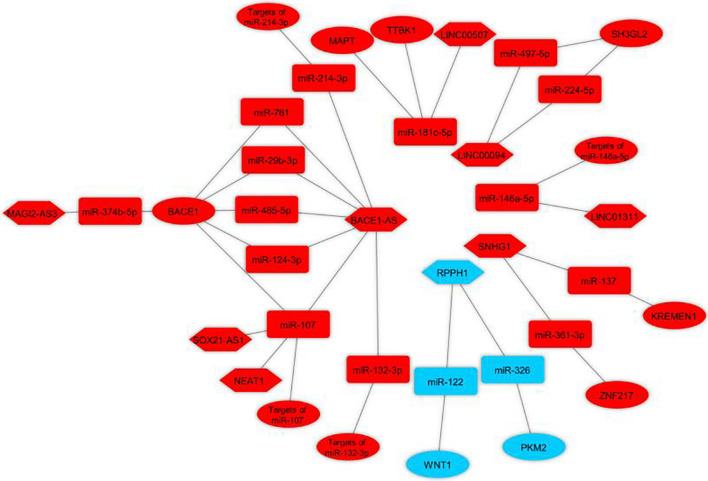
Validated lncRNA-associated ceRNA axes in Alzheimer’s disease. The loops were visualized using Cytoscape v3.8.0 software (Shannon et al., 2003) based on lncRNA-miRNA-mRNA interactions. Red represents the damaging role, and blue represents the protective role of competing endogenous RNA axes. LncRNAs, miRNAs, and mRNAs are represented by hexagon, round rectangle, and ellipse, respectively. LncRNA, long non-coding RNA; miRNA, microRNA.

**FIGURE 4:**
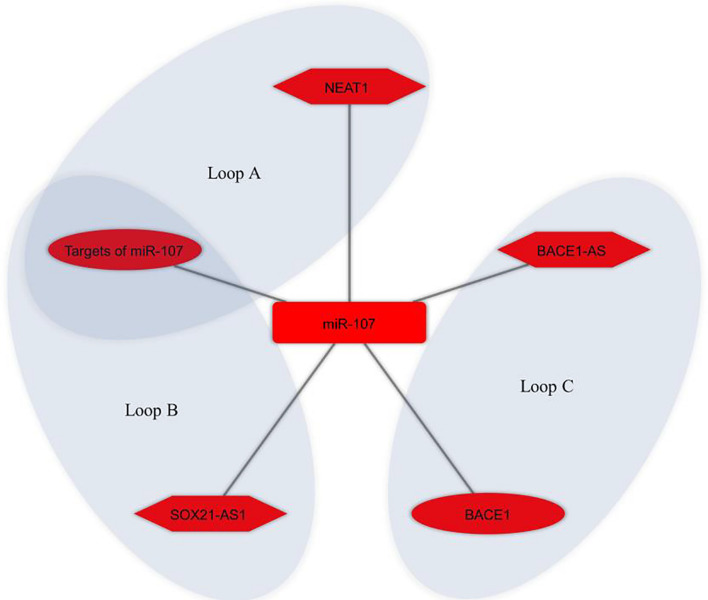
*miR-107* regulates different competing endogenous RNA (ceRNA) loops. Loop A (*NEAT1* and targets of *miR-107*), loop B (*SOX21-AS1* and targets of *miR-107*), and loop C (*BACE1-AS* and *BACE1*) were all regulated by *miR-107*. Red represents the damaging role of ceRNA axes. Lon non-coding RNAs, microRNAs, and mRNAs are represented by hexagon, round rectangle, and ellipse, respectively.

Bioinformatics analysis was used to identify the potential ceRNA interactions. Also, different molecular techniques were utilized to validate the components of the ceRNA loop and investigate their involvement in AD pathogenesis. The reported loops are shown in Figure 3. The lncRNA BACE1-antisense (*BACE1-AS*) was reported in four studies (Faghihi et al., 2010; Zeng et al., 2019; Ge et al., 2020; He W. et al., 2020), two of which had *BACE1-AS*/*miR-485-5p*/*BACE1* regulatory axis (Faghihi et al., 2010; Zeng et al., 2019), as well as *BACE1* was a target mRNA in three studies (Faghihi et al., 2010; Zeng et al., 2019; Zhang and Wang, 2021). The lncRNA small nucleolar RNA host gene 1 (*SNHG1*) (Wang et al., 2019; Gao et al., 2020) and the lncRNA ribonuclease P RNA component H1 (*RPPH1*) (Gu et al., 2020, 2021) were each reported in two articles. The remaining ceRNAs were reported once each in six studies (Ke et al., 2019; Zhu et al., 2019; Yan et al., 2020; Yue et al., 2020; Fan et al., 2021; Zhang and Wang, 2021). Among the identified miRNAs, *miR-107* was reported in three regulatory axes in three different studies (Ke et al., 2019; Zeng et al., 2019; Yue et al., 2020). According to these results, we found that three independent ceRNA interaction loops (loop A, loop B, and loop C) are regulated by *miR-107*. Loop A includes nuclear enriched abundant transcript 1 (*NEAT1*) and targets of *miR-107*, loop B includes *SOX21* antisense RNA1 (*SOX21-AS1*) and targets of *miR-107*, loop C includes *BACE1-AS* and *BACE1* (Figure 4).

## Discussion

The most contentious issue with ceRNA regulation is whether or not it is effective in physiological contexts. Most studies questioning ceRNA functions reach the conclusion that regulation of ceRNA is highly improbable to yield biologically major impacts at physiological RNA concentrations; however, these experiments cannot rule out the potential of potent miRNA sponges or the marked downregulation or upregulation of ceRNAs and miRNAs at particular developmental stages or subcellular sites (Denzler et al., 2014; Cai and Wan, 2018). There are many reports indicating that ceRNA machinery operates in numerous diseases and that ceRNA is expressed differently with various tissues, cells, and subcellular situations. The literature represents novel visions by which designing ceRNA-mechanism-based therapeutical utilization can be facilitated for the manipulation of special developmental phases and disease pathogenicity with the use of synthesized oligonucleotides specific to sequences (Khvorova and Wolfson, 2012). Over the last decade, significant effort has been put toward the clinical use of RNA-based therapies, mainly using antisense oligonucleotides as well as small interfering RNAs, with many obtaining Food and Drug Administration (FDA) approval (Winkle et al., 2021). However, trial findings have been mixed, with some research claiming strong benefits and others showing minimal effectiveness or toxicity. Alternate entities, including lncRNA-based therapies, are gaining interest (Winkle et al., 2021). The lncRNAs’ wide function offers many possibilities for clinical application, the methods of which must be adapted to the lncRNA’s mode of action (Arun et al., 2018). Investigating natural antisense transcripts (NATs): lncRNAs that are transcribed from opposite strands of adjacent genes and negatively regulate them in cis is an intriguing discovery. AntagoNATs are ASOs that target NATs and have demonstrated extremely promising preclinical findings for gene reactivation in the central nervous system (CNS) (Winkle et al., 2021). AntagoNATs effectively increased the expression of brain-derived neurotrophic factor (BDNF), a protein important in memory development (Modarresi et al., 2012), and the healthy allele of *SCN1A*, the haploinsufficiency of which triggers Dravet syndrome (Hsiao et al., 2016). Remarkably, *BDNF-AS*-targeting antagoNATs were effectively delivered across the blood–brain barrier in a murine model utilizing a minimally invasive nasal depot (MIND). MIND is a medication delivery system that directs drug administration to the olfactory submucosal region and has achieved about 40% effectiveness compared to riskier invasive delivery methods (Padmakumar et al., 2021). Such encouraging findings imply that lncRNA-based therapies will soon enter clinical testing. Moreover, the therapeutical impact of ASO-based lncRNA knockdown has shown promise in patients with Angelman syndrome, a single-gene illness represented by intellectual disability (Bird, 2014; Meng et al., 2015). Despite no development of ASOs that target lncRNA in the therapy of NDDs, ASOs that target mRNAs have received approval by the FDA for the therapy of Duchenne muscular dystrophy and spinal muscular atrophy (Wurster and Ludolph, 2018). Furthermore, ASOs have presented potential effectiveness in the therapy of Huntington’s disease (HD) by affecting the huntingtin gene (*HTT*) as the target, in the therapy of amyotrophic lateral sclerosis by influencing *SOD1* and *C9ORF72* as the targets, and in the therapy of AD by affecting *MAPT* (tau) as the target (Scoles et al., 2019). Hence, lncRNAs and lncRNA-associated ceRNA axes can also serve as a new therapeutical target for AD therapy. In this study, we performed a scoping review and identified nine lncRNAs that were experimentally validated to act as ceRNA in AD in human studies. These lncRNAs are discussed below.

### BASE1-AS

As mentioned above, in AD patients, the extracellularly deposited plaque of the Aβ peptide is one of the representative pathologic signs. To produce Aβ, APP needs to be sequentially cleaved proteolytically with β-secretase and γ-secretase. BACE1 is a critical enzyme in AD pathophysiology responsible for Aβ plaque formation through the cleavage of APP in collaboration with γ-secretase. Thus, a misregulated BACE1 can have a major contribution to this pathologic event. Both miRNAs and lncRNAs have been involved in regulating *BACE1* post-transcriptionally, with which lncRNA *BACE1-AS* has the utmost relevancy (Zeng et al., 2019). The lncRNA *BACE1-AS* was the first validated component of a ceRNA loop described in AD pathogenesis. Both *BACE1-AS* and *BACE1* mRNA transcripts originate from a similar locus in chromosome 11 in humans; the transcription of *BACE1* mRNA and *BACE1-AS* is triggered from the sense and antisense strands, respectively. An RNA duplex is formed by pairing *BACE1-AS* to *BACE1*, leading to a structurally changed *BACE1* and improved stableness of mRNA. Consequently, *BACE1-AS* has a role in increasing both the mRNA and protein concentrations of *BACE1* (Faghihi et al., 2008, 2010). This ceRNA pair had been reported in two studies. The first loop was *BACE1-AS*/*miR-485-5p*/*BACE1*, which was shown to be involved in AD pathogenesis through *BACE1* post-transcriptional regulation (Faghihi et al., 2010). It was also shown that *BACE1-AS* and to a lower level *BACE1* were up-regulated, and the expression of *miR-485-5p* was decreased in the brain of AD patients (Faghihi et al., 2010). The second study showed that *BACE1-AS* prevented *BACE1* mRNA degradation by sponging *miR-29b-3p*/*miR-107*/*miR-124-3p*/*miR-485-5p*/*miR-761* in the pathophysiology of AD. In addition to the mentioned ceRNA pair, it was shown that *BACE1-AS* exacerbated isoflurane (anesthetic)-induced neurotoxicity by *BACE1-AS*/*miR-214-3p* axis in AD (He W. et al., 2020). This drug increases the risk of AD by increasing Aβ production and its oligomerization, as well as neuronal apoptosis (Xie et al., 2006, 2008; Xie and Xu, 2013). It is noteworthy that increased expression of *BACE1-AS* and down-regulation of *miR-214-3p* in the plasma samples of AD patients was also reported. In addition, *BACE1-AS* is a ceRNA for miR-132-3p. This axis was involved in the berberine-mediated neuroprotective effect in AD (Ge et al., 2020). *Berberine* is an isoquinoline alkaloid found in many medicinally important plants (Kumar et al., 2015). Berberine has been reported to play a crucial role in AD treatment (Yuan et al., 2019).

### MAGI2 Antisense RNA 3 (*MAGI2-AS3*)

Alongside *BACE1-AS*, *MAGI2-AS3* (via *miR-374b-5p*) could reportedly regulate *BACE1* mRNA levels, Aβ-induced neurotoxicity, and neuroinflammation in AD (Zhang and Wang, 2021). This underlines that the *BACE1* is complexly regulated in AD by the mediation of multiple ceRNA networks. It was also shown that serum *MAGI2-AS3* and *miR-374b-5p* expression was significantly up-regulated and down-regulated in AD patients compared with healthy controls, respectively (Zhang and Wang, 2021). LncRNA *MAGI2-AS3* is reportedly a sponger of *miR-374b-5p* in ovarian carcinoma and hepatocellular carcinoma (Gokulnath et al., 2019; Yin Z. et al., 2019). It has been found to be involved in regulating cell survivability in a variety of diseases (Cao et al., 2020; He J. et al., 2020). There are also reports on the association between *MAGI2-AS3* and chronic inflammatory illnesses (Liao et al., 2020).

### SNHG1

In AD pathology, *SNHG1* involves in Aβ-induced neuronal injury via two different ceRNA regulatory axes, *SNHG1*/*miR-137*/kringle containing transmembrane protein 1 (*KREMEN1*) (Wang et al., 2019) and *SNHG1*/*miR-361-3p*/zinc finger gene 217 (*ZNF217*) (Gao et al., 2020). *SNHG1* has been well-studied in different types of cancers due to its oncogenic role (Huang et al., 2018). It is shown that it can also promote neuronal autophagy and neuroinflammation in Parkinson’s disease (PD) (Cao et al., 2018; Chen et al., 2018b). *KREMEN1* encodes a receptor for Dickkopf (DKK) proteins that functionally cooperates with DKK1/2 to inhibit wingless (WNT)/beta-catenin signaling (Mulvaney et al., 2016; Stelzer et al., 2016). It exerts its pro-apoptotic activity in a Wnt-independent pathway (Causeret et al., 2016). It has been shown that the silencing of *KREMEN1* prevents Aβ-mediated synapse loss in AD (Ross et al., 2018). Besides, the oncogenic role of *ZNF217* has been shown in many cancers (Quinlan et al., 2007). In AD studies, the down-regulation of *ZNF217* could relieve Aβ-induced neurotoxicity (Wang J. et al., 2018). It had also been shown that the neuroprotective effect of resveratrol could occur through *SNHG1*/*miR-361-3p*/*ZNF217* axis (Gao et al., 2020). Resveratrol is a polyphenolic compound that its high concentrations are found in red grapes, blueberries, and peanuts (Evans et al., 2017). Various studies have proved its antioxidant and anti-inflammatory effects on AD (Gomes et al., 2018; Corpas et al., 2019; Kong et al., 2019).

### NEAT1

*NEAT1* aggravated Aβ-induced neuronal injury via acting as a sponge for *miR-107* (Ke et al., 2019). Knockdown of NEAT1 attenuated Aβ induced inhibition of viability and promotion of apoptosis and p-tau levels. MiR-107 was down-regulated, and it reversed Aβ-induced injury when overexpressed in Aβ-treated cells (Ke et al., 2019). *NEAT1* is frequently overexpressed in human tumors and proposed as a novel target for human cancer therapy and diagnosis by sponging miRNAs (Dong et al., 2018). According to previous studies, it also has a crucial role in HD and PD (Chanda et al., 2018; Liu and Lu, 2018). In AD studies, its increased expression levels were shown in brain tissues of AD patients, compared to controls (Spreafico et al., 2018).

### SOX21-AS1

*SOX21-AS1*, similar to *NEAT1*, exacerbated Aβ-induced neuronal injury via sequestering *miR-107* (Yue et al., 2020). *SOX21-AS1* is a recently discovered lncRNA that can suppress neuronal apoptosis of hippocampal cells and mitigate oxidative stress in AD, hence, being involved as an agent in AD pathogenicity (Zhang L. et al., 2019).

It is noteworthy that, as mentioned above, the present study identified that *miR-107* was at the center of three different loops, as shown in Figure 4, which suggests it is a key miRNA for both biological researches and ceRNA-based therapeutic purposes in AD studies. The *miR-107* family is a group of evolutionarily conserved miRNAs that show high expression in the human cerebral cortex. Numerous studies have shown misregulation of this miRNA in AD brains (Wang et al., 2008; Gupta et al., 2017; Moncini et al., 2017). It has been reported that it may have a protective role in AD by preventing Aβ-induced blood–brain barrier (BBB) disruption, endothelial cell dysfunction (Liu et al., 2016), and Aβ-induced neuronal damage (Jiao et al., 2016; Shu et al., 2018; Chen et al., 2020). *miR-107* shows a certain potential to be used as a biomarker in AD (Herrera-Espejo et al., 2019; Prendecki et al., 2019; Swarbrick et al., 2019). The ceRNA interactions are mediated by miRNAs, and altered miRNAs expression result in dysregulation of its competitive interactors. On the other hand, it is hypothesized that ceRNAs in the modules play a role in disease progression as a whole instead of acting individually (Chen et al., 2018a). Thus, these identified ceRNA modules comprising *NEAT1*, *SOX21-AS1*, *BACE1-AS*, *miR-107*, targets of *miR-107*, and *BACE1* can be further assessed as potential ceRNA modules for ceRNA-based therapeutic purposes in AD.

### Long Intergenic Non-protein Coding RNA 507 (*LINC00507*)

It was reported that *LINC00507*/*miR-181c-5p*/tau-tubulin kinase-1 (*TTBK1*)/*MAPT* axis regulated tau hyperphosphorylation via P25/P35/GSK3β signaling pathway (Yan et al., 2020). LincRNAs are a subclass of lncRNAs. *LINC00507* has an age-dependent expression pattern and is specifically expressed in the primate cortex (Mills et al., 2016). *MAPT* encodes the tau protein, and TTBK1 is a CNS-specific protein kinase that involves in tau hyper-phosphorylation and deposition in AD (Ikezu and Ikezu, 2014). It also has been shown that P25/P35/GSK3β signaling pathway deteriorates tauopathy (Noble et al., 2003).

### Long Intergenic Non-protein Coding RNA 94 (*LINC00094*)

As indicated in previous reports, LncRNA *LINC00094* (called *BRD3OS* as well) is involved in regulating BBB penetrability in the AD microenvironment by sponging *miR-224-5p* and *miR-497-5p*, and *SH3GL2* mRNA is targeted by both of them (Zhu et al., 2019). *LINC00094* may reportedly act as a prognostic biomarker of lung cancer (Li et al., 2017). Additionally, microarray examination revealed the *LINC00094* down-regulation in Memantine-incubated cells. As an *N*-methyl-D-aspartate (NMDA) receptor antagonist, memantine has received wide applications for AD treatment (Zhu et al., 2019). *SH3GL2* encodes Endophilin-1, an endocytosis protein that has a marked increase in the AD brain and is responsible for Aβ-induced postsynaptic dysfunction (Yin Y. et al., 2019).

### Long Intergenic Non-protein Coding RNA 1311 (*LINC01311*)

It was reported that the *LINC01311*/*hsa-miR-146a-5p* axis could operate as a functional regulator in AB1-42-stimulated apoptosis, proliferation slowdown, autophagy, and accumulated APP in human-lineage neurons (Fan et al., 2021). As a new lncRNA, *LINC01311* was discovered throughout human genome-wide screening, which has an aberrant expression in human liver and prostate cancers (Zhu et al., 2016; Imada et al., 2020). However, the functional activity of *LINC01311* has never been clarified in other human disorders.

### RPPH1

Unlike the lncRNAs discussed above, *RPPH1* apparently exerts a neuroprotective compensatory mode of action in AD pathology via two varying ceRNA axes: *RPPH1*/*miR-122*/*WNT1* (Gu et al., 2020) and *RPPH1*/*miR-326*/pyruvate kinase M2 (*PKM2*) (Gu et al., 2021). Specifically, evidence indicates that it can mitigate Aβ25–35-stimulated neuronal damage, apoptosis, and endoplasmic reticulum stress (Gu et al., 2020, 2021). *RPPH1* is the RNA component of RNase P, which plays a role in tRNA maturation in Archaea, Bacteria, and Eukarya (Evans et al., 2006). Wnt/β-catenin signaling has a confirmed essential function in developing AD. Huperzine A, which reversibly and selectively inhibits acetylcholinesterase and is utilized for AD treatment, has been reported to have a neuroprotecting impact by activating Wnt/β-catenin signaling in AD (Wang et al., 2011). As shown previously, the BBB failure in AD is caused by an impaired Wnt/β-catenin signaling (Liu et al., 2014). Besides, research indicates that activating the Wnt/β-catenin signaling is capable of protecting neuronal cells by regulating survival and c-myc, as well as apoptosis-linked proteins Bcl-2 and Bax (Jeong et al., 2014). As a glycolytic sensor, PKM2 has a crucial contribution to the dephosphorylation of phosphoenolpyruvate to pyruvate and catalysis of the final stage of glycolysis (Nakatsu et al., 2015). The emergence of recent documentation has highlighted that *PKM2* is involved in AD. According to reports, oxidatively inactivated PKM2 had an association with the progress of AD from mild cognitive impairment (Butterfield et al., 2006). The poly(ADP-ribose)polymerase 1 could also modulate PKM2, indicating that the PKM2-linked glycolytic pathway has a contribution to AD (Martire et al., 2016).

### Limitations of Scoping Review

A scoping review is different from a systematic review. It systematically studies the literature, quantitatively synthesizes the accomplishments and sums up the gaps in a particular field’s literature rather than evaluating it to offer a solution to a particular question. The approach is not substantially different from a systematic review, but an evaluation of methodological limitations or risk of bias of the evidence contained in a scoping review is not usually undertaken. A scoping review is often conducted before a systematic review to examine the literature and determine pertinent research topics to be addressed by a subsequent systematic review. Additionally, a scoping review does not seek to aggregate results via meta-analysis but rather maps the literature to determine themes, gaps, and patterns (Arksey and O’Malley, 2005; Levac et al., 2010). Because ceRNAs and AD is relatively new, a scoping review was conducted to evaluate the extent, scope, and type of research effort in this field to bring attention to topics where further study is required. Our results, however, should be interpreted with caution because we decided to perform a scoping review, which omits quality evaluations of the articles. The most significant limitation of this study is most likely the dearth of evidence available for review.

## Conclusion

LncRNA-associated ceRNA regulation produces biologically significant effects in various diseases so it can elucidate the pathogenic procedures and offer options for new therapies. Thus, our efforts to understand different aspects of ceRNA regulatory mechanisms in AD pathogenesis provide new insights into the potential molecular targets, discover ceRNA-based biomarkers, and design ceRNA-based therapeutic applications.

## Data Availability Statement

The original contributions presented in the study are included in the article/supplementary material, further inquiries can be directed to the corresponding authors.

## Author Contributions

MT, MR, and HS wrote the draft and revised it. MA, YD, OR, AJ, and NA collected the data and designed the tables and figures. All authors read the draft and approved the submitted version.

## Conflict of Interest

The authors declare that the research was conducted in the absence of any commercial or financial relationships that could be construed as a potential conflict of interest.

## Publisher’s Note

All claims expressed in this article are solely those of the authors and do not necessarily represent those of their affiliated organizations, or those of the publisher, the editors and the reviewers. Any product that may be evaluated in this article, or claim that may be made by its manufacturer, is not guaranteed or endorsed by the publisher.
